# Amyloid β-protein oligomers upregulate the β-secretase, BACE1, through a post-translational mechanism involving its altered subcellular distribution in neurons

**DOI:** 10.1186/s13041-015-0163-5

**Published:** 2015-11-09

**Authors:** Naomi Mamada, Daisuke Tanokashira, Ai Hosaka, Fuyuki Kametani, Akira Tamaoka, Wataru Araki

**Affiliations:** Department of Demyelinating Disease and Aging, National Institute of Neuroscience, NCNP, Kodaira, Tokyo 187-8502 Japan; Department of Neurology, Faculty of Medicine, University of Tsukuba, Tsukuba, Ibaraki 305-8575 Japan; Department of Dementia and Higher Brain Function, Tokyo Metropolitan Institute of Medical Science, Setagaya, Tokyo 156-8506 Japan

**Keywords:** Alzheimer’s disease, Amyloid β-protein, BACE1, Neuron, Oligomer

## Abstract

**Background:**

β-Site amyloid precursor protein cleaving enzyme 1 (BACE1) is a membrane-bound aspartyl protease that initiates amyloid β-protein (Aβ) generation. Aberrant elevation of BACE1 levels in brains of Alzheimer’s disease (AD) patients may involve Aβ. In the present study, we used a neuron culture model system to investigate the effects of Aβ on BACE1 expression as well as the underlying mechanisms.

**Results:**

Rat primary cortical neurons were treated with relatively low concentrations (2.5 μM) of Aβ42 oligomers (Aβ-O) or fibrils (Aβ-F) for 2–3 days. Aβ-O induced a significant increase in protein levels of BACE1, while Aβ-F only had a marginal effect. Levels of amyloid precursor protein (APP) and the major α-secretase, ADAM10, remained unaltered upon treatment with both types of Aβ. Aβ-O treatment resulted in activation of eIF2α and caspase 3 in a time-dependent manner, with no changes in the endoplasmic reticulum (ER) stress marker, GRP78, indicating that a typical ER stress response is not induced under our experimental conditions. Furthermore, Aβ-O did not affect BACE1 mRNA expression but augmented the levels of exogenous BACE1 expressed via recombinant adenoviruses, indicating regulation of BACE1 protein expression, not at the transcriptional or translational but the post-translational level. Immunocytochemical analysis revealed that Aβ-O causes a significant increase in BACE1 immunoreactivity in neurites (both axons and dendrites), but not soma of neurons; this change appears relevant to the mechanism of Aβ-O-induced BACE1 elevation, which may involve impairment of BACE1 trafficking and degradation. In contrast, Aβ-O had no effect on APP immunoreactivity.

**Conclusion:**

Our results collectively suggest that Aβ oligomers induce BACE1 elevation via a post-translational mechanism involving its altered subcellular distribution in neurons, which possibly triggers a vicious cycle of Aβ generation, thus contributing to the pathogenetic mechanism of AD.

**Electronic supplementary material:**

The online version of this article (doi:10.1186/s13041-015-0163-5) contains supplementary material, which is available to authorized users.

## Background

Alzheimer’s disease (AD) is the most common neurodegenerative dementia characterized by two major pathological hallmarks, extracellular amyloid plaques and intracellular neurofibrillary tangles composed of amyloid β-protein (Aβ) and phosphorylated tau protein, respectively [1]. The finding that mutations in familial AD genes (amyloid precursor protein (APP) and presenilins) affect Aβ generation to increase total Aβ or the Aβ42/40 ratio supports the hypothesis that Aβ accumulation plays a central role in AD pathology [2]. The longer form of Aβ, Aβ42, is more aggregable and probably more pathogenic than Aβ40. Recent evidence further suggests that Aβ oligomers, soluble aggregated forms of Aβ, act as an initiator of AD by inducing the development of tau pathology and synaptic dysfunction [3, 4]. While the exact toxic forms of Aβ oligomers and mechanisms underlying their neurotoxicity remain elusive at present, Aβ oligomers are considered a viable therapeutic target for AD [3, 4].

Aβ is a cleavage product of APP by two proteases, specifically, β-site APP cleaving enzyme 1 (BACE1) and the γ-secretase complex. APP is initially cleaved by BACE1, producing secreted APP-β and C-terminal fragment (CTF) known as β-CTF. β-CTF is subsequently cleaved by γ-secretase to release Aβ. Alternatively, APP is cleaved by α-secretase, mainly constituting ADAM10, at the α-site within the Aβ sequence, precluding Aβ formation [5]. BACE1 is a type I transmembrane aspartyl protease abundantly expressed in neurons in the brain [6]. As a rate-limiting enzyme for Aβ production, BACE1 is considered one of the major therapeutic targets for AD [7]. The protease is matured through the Golgi apparatus and targeted to the plasma membrane where it is rapidly internalized to endosomes. As BACE1 activity is optimal at acidic pH, cleavage of APP occurs predominantly in the endosome. BACE1 is cycled between the plasma membrane, endosomes and the *trans*-Golgi network (TGN), and mainly degraded in lysosomes [7, 8].

BACE1 levels and activity are increased in the brains of AD patients [9–15] and mouse models of AD [15–17], supporting the involvement of aberrant BACE1 in AD pathogenesis. In addition, BACE1 expression is reported to be increased under various stress conditions, such as ischemia [18, 19]. However, the mechanism underlying BACE1 increase in AD remains to be defined. It is uncertain whether a direct relationship exists between Aβ and BACE1, although data supporting an association have recently been described [16]. To elucidate this issue, we investigated the effects of Aβ (both oligomer and fibril forms) on BACE1 expression as well as the underlying mechanisms using a primary neuron culture system. Our current findings collectively demonstrate that Aβ oligomers induce BACE1 elevation via a post-translational mechanism involving its altered subcellular distribution.

## Results

### BACE1 protein levels are increased upon Aβ42 oligomer treatment

**Fig. 1:**
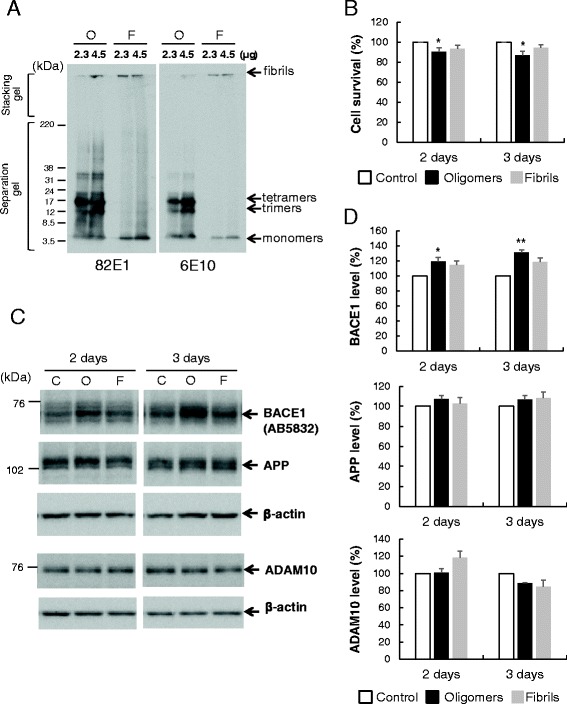
Aβ oligomers specifically enhance the BACE1 protein level in primary neurons. **a** Aβ42 oligomers (O) or fibril (F) preparations (2.3 or 4.5 μg) were separated with the Tris/Tricine gel, followed by Western blot analysis with anti-Aβ antibodies, 82E1 or 6E10. **b** Neurons grown on a 12-well plate were treated with 2.5 μM Aβ as above followed by cell survival assay using the Cell Counting Kit-8, as described in Methods. Relative levels of cell survival are presented as a graph. Data represent means ± SEM from four separate experiments. **c** Primary cortical neurons (9DIV) grown on a six-well plate were treated with 2.5 μM Aβ oligomers (O), fibrils (F) or vehicle (C) for 2 or 3 days, followed by Western blot analysis with antibodies against BACE1, APP, ADAM10 or β-actin. **d** Quantitative analysis of BACE1, APP and ADAM10 levels after normalization to β-actin. Data represent means ± SEM from three or four separate experiments. *p < 0.05, **p < 0.01, compared with control

Aβ42 oligomers (Aβ-O) and fibrils (Aβ-F) were prepared using previously established protocols [20]. Western blot analysis revealed that the Aβ-O preparation consists mainly of trimers and tetramers in addition to monomers and oligomers with molecular sizes of 30–40 kDa, while Aβ-F is composed of monomers and aggregates that do not enter the stacking gel (Fig. 1a). Comparable data were obtained with the two Aβ antibodies, 82E1 and 6E10. However, 82E1 detected oligomers with higher molecular sizes more clearly than 6E10.

In designing an experimental protocol, we attempted to achieve a condition whereby Aβ treatment induces relatively modest neuronal damage, mimicking physiological conditions. In our pilot experiments, neurons displayed relatively modest responses to 2.5 μM Aβ-O, which was therefore employed for further experiments.

To estimate the effects of Aβ on cell survival, the cell survival assay was performed using Cell Counting Kit-8. Cell survival remained almost unchanged upon treatment with Aβ-F, and was only very slightly (10–15 %) decreased upon Aβ-O treatment on days 2 and 3 (Fig. 1b) compared with control, suggesting that Aβ induces almost no apparent cell death under our experimental conditions.

**Fig. 2:**
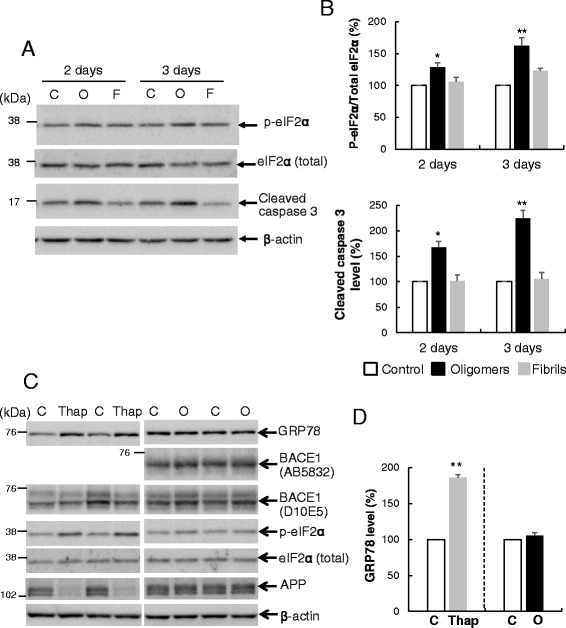
Aβ oligomers induce activation of caspase 3 and eIF2α, but do not alter GRP78 levels. **a** Primary neurons were treated with 2.5 μM Aβ oligomers (O), fibrils (F) or vehicle (C) for 2 or 3 days, followed by Western blot analysis of cell lysates. Membranes were probed with the indicated antibodies. **b** Quantitative analysis of p-eIF2α/total eIF2α ratios and cleaved caspase 3 levels. Data represent means ± SEM from three separate experiments. *p < 0.05 and **p < 0.01, compared with control. **c** Primary neurons were treated with 2.5 μM Aβ oligomers (O) for 3 days or 1 μM thapsigargin (Thap) for 1 day, followed by Western blot as above. Membranes were probed with the indicated antibodies. **d** Quantitation of GRP78 levels. Data represent means ± SEM from three independent experiments. **p < 0.01, compared with control

Next, we examined the effects of Aβ on BACE1 protein levels. Primary cortical neurons were treated with 2.5 μM Aβ-O, Aβ-F or vehicle for 2 or 3 days, followed by Western blot analysis. Endogenous BACE1 levels in neurons treated with Aβ-O for 2 or 3 days were significantly increased by 20 and 31 %, respectively, compared with those in control (Fig. 1c, d). Aβ-F also induced a slight increase in BACE1 protein, but the effect was relatively weaker than Aβ-O. Data obtained with two different BACE1 antibodies were almost identical, as shown in Fig. 2c. In contrast, APP protein levels in neurons treated with Aβ-O or Aβ-F were not significantly altered, compared with those in control neurons (Fig. 1c, d). Similarly, Aβ-O and Aβ-F did not affect ADAM10 levels (Fig. 1c, d). Thus, Aβ-O specifically acts to increase BACE1 protein levels without inducing alterations in APP and ADAM10.

### Aβ oligomers activate caspase 3 and eIF2α

Since Aβ-O induces various cellular responses, including caspase activation, we analyzed its effects on proteins related to apoptotic and other stress responses. First, we examined cleaved caspase 3 (the activated form), an executor molecule in the apoptotic cascade. Levels of cleaved caspase 3 were significantly increased in neurons treated with Aβ-O compared with controls, in a time-dependent manner to 167 and 225 % on days 2 and 3, respectively (Fig. 2a, b). Next, we examined phosphorylated eIF2α (p-eIF2α), an activated form of the translation initiation factor, eIF2α, that plays an important role in various stress response pathways. The p-eIF2α/total eIF2α ratio was significantly increased by 29 and 62 % in Aβ-O-treated neurons, compared with control, on days 2 and 3, respectively (Fig. 2a, b). In contrast, levels of cleaved caspase 3 and p-eIF2α/total eIF2α remained almost unaltered in neurons treated with Aβ-F, compared with controls. As p-eIF2α is elevated in endoplasmic reticulum (ER) stress, we further examined the ER stress marker, GRP78. Thapsigargin was used as a positive control for ER stress. Levels of GRP78 and p-eIF2α/total eIF2α were significantly increased in neurons treated with thapsigargin while GRP78 remained unchanged in those treated with Aβ-O (Fig. 2c, d). Moreover, in contrast to Aβ-O, thapsigargin suppressed APP and BACE1 levels in neurons (Fig. 2c, d). Clearly, the changes induced by Aβ-O do not result from typical ER stress response in our experimental conditions.

### Aβ oligomers induce BACE1 upregulation at the post-translational level

**Fig. 3:**
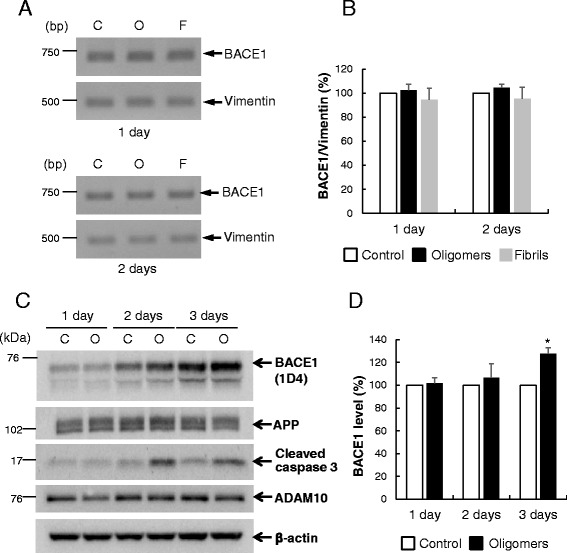
Aβ oligomers augment BACE1 levels, not at the transcriptional or translational, but the post-translational level. **a** Total RNA was extracted from neurons treated with 2.5 μM Aβ oligomers (O), fibrils (F) or vehicle (C) for 1 or 2 days, followed by semi-quantitative RT-PCR. **b** Band intensities of BACE1 and vimentin in (A) were quantified, and BACE1 mRNA levels normalized to those of vimentin presented as a graph. Data represent means ± SEM from three separate experiments. **c** Primary cortical neurons (8DIV) were infected with recombinant BACE1 adenoviruses expressing rhodopsin-tagged BACE1. The next day, cells were exposed to Aβ oligomers for 1–3 days, followed by Western blot with anti-rhodopsin tag 1D4. Membranes were reprobed with the different antibodies specified. **d** Quantitative analysis of exogenous BACE1 levels. Data represent means ± SEM from three independent experiments. *p < 0.05, compared with control

To ascertain the molecular mechanism underlying the Aβ-O-induced increase in BACE1 protein, BACE1 mRNA levels were assessed in neurons treated with Aβ using semi-quantitative RT-PCR. BACE1 mRNA levels normalized by vimentin were not significantly altered in neurons treated with Aβ-O or Aβ-F at days 1 and 2 (Fig. 3a, b), compared with control neurons. These data suggest that Aβ-O-induced elevation of BACE1 protein is not attributable to alterations in mRNA expression.

Next, we clarified whether the increase in BACE1 protein levels induced by Aβ-O is translationally regulated. To this end, we investigated the effects of Aβ-O on exogenously expressed BACE1. Primary cortical neurons were infected with recombinant adenoviruses expressing human BACE1 with a C-terminal rhodopsin tag [21], and after 1 day, infected cells were treated with Aβ-O for 1–3 days. Western analysis with anti-rhodopsin tag antibody (1D4) disclosed a significant increase in exogenous BACE1 levels in Aβ-O-treated neurons on day 3 (by ~25 %), compared with control (Fig. 3c, d). Cleaved caspase 3 levels were additionally increased in Aβ-O-treated neurons on days 2 and 3, while levels of APP (~95 % of control) and ADAM10 (~103 % of control) remained unaffected on day 3 (Fig. 3c). Since exogenous BACE1 is regulated under the CAG promoter and does not contain 5’UTR, which is known to be translationally regulated by p-eIF2α [22], Aβ-O-induced BACE1 elevation appears independent of translational regulation via p-eIF2α. The collective data suggest that the Aβ-O-induced increase in BACE1 protein occurs through a post-translational mechanism.

### Aβ oligomer affects the subcellular distribution of BACE1

**Fig. 4:**
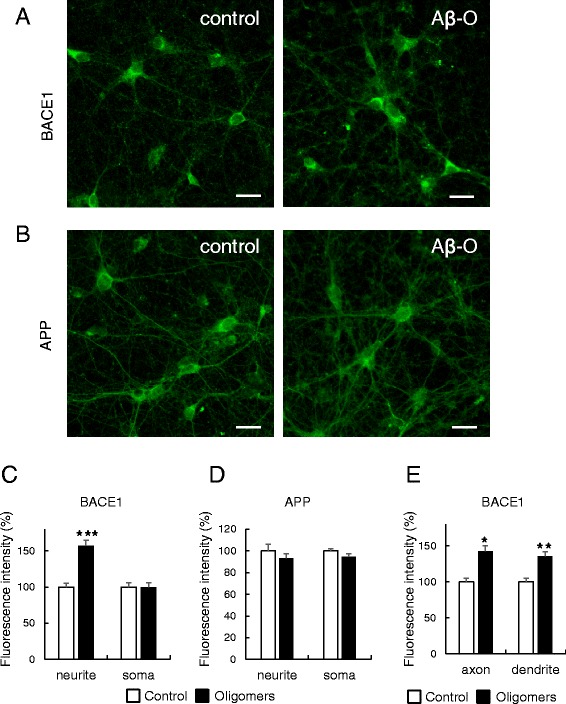
Immunocytochemical analysis of BACE1 and APP. **a**, **b** Primary cortical neurons grown on coverslips were treated with 2.5 μM Aβ oligomers (Aβ-O) for 3 days, followed by immunofluorescence staining with anti-BACE1 (**a**) or anti-APP (**b**) antibodies. Immunostaining of control and Aβ-O-treated cells was carried out under the same conditions, and their images were acquired at the same exposure time. Scale bar = 20 μm. Intensity of BACE1 immunoreactivity is relatively higher in neurites, but not soma of neurons treated with Aβ-O than control. **c**, **d** Fluorescence intensities of BACE1 (**c**) or APP (**d**) in soma and neurites were separately quantified as described in Methods, and the relative levels depicted on a graph. (*n* = 18 ~ 20, ***p < 0.001). **e** Fluorescence intensities of BACE1 in axons and dendrites were separately quantified as above in specimens doubly immunostained with BACE1 and MAP2, and the relative levels depicted on a graph (*n* = 24, *p < 0.05, **p < 0.01)

To gain further insights into the mechanism underlying Aβ-O-induced elevation of BACE1, we analyzed the influence of Aβ-O on intraneuronal localization of BACE1 using immunocytochemistry. In control untreated neurons, endogenous BACE1 immunoreactivity was observed in neuronal soma and neurites, consistent with recent findings [23, 24]. Interestingly, in neurons treated with Aβ-O for 3 days, the intensity of BACE1 immunoreactivity was higher relative to control, although levels in soma were comparable between control and Aβ-O-treated neurons (Fig. 4a, c). Further double immunofluorescence staining with BACE1 and microtubule-associated protein 2 (MAP2) antibodies revealed that Aβ-O treatment triggers increased BACE1 immunoreactivity in both axons and dendrites (Fig. 4e). To determine whether this change in intracellular localization is specific for BACE1, we subsequently analyzed the effect of Aβ-O on APP distribution. Our data revealed diffuse localization of APP in soma and neurites, with no significant differences in immunoreactivity between control and Aβ-O-treated neurons (Fig. 4b, d). In addition, double immunofluorescence staining with anti-BACE1 and anti-APP indicated partial colocalization of their immunoreactivities in both soma and neurites, the extent of which tended to be increased in Aβ-O-treated neurons relative to control (Additional file 1: Figure S1). In view of these findings, we propose that Aβ-O specifically affects the subcellular distribution of BACE1 to augment its levels in neuritic compartments.

It is possible that Aβ-O may induce BACE1 accumulation in neurites by altering BACE1 degradation via lysosomal pathway, since BACE1 is suggested to be primarily degraded via this pathway [7, 8, 23]. We therefore examined whether lysosomal disturbance leads to abnormal subcellular distribution of BACE1 by analyzing BACE1 levels and immunoreactivities in primary neurons treated with a lysosomotropic agent chloroquine for 1 day. Western blot analysis showed that BACE1 protein levels were significantly increased (~40 %) in chloroquine-treated neurons, compared with control (Additional file 2: Figure S2A). Immunocytochemical analysis revealed that the intensity of BACE1 immunoreactivity was increased significantly in both soma and neurites of chloroquine-treated neurons, compared with control (Additional file 2: Figure S2B). Quantitatively, the extent of BACE1 increment was greater in neurites (~70 %) than in soma (~40 %) (Additional file 2: Figure S2C). These results suggest that perturbed lysosomal degradation results in BACE1 elevation in neurites as well as soma.

Finally, we examined whether APP processing is affected by treatment with Aβ-O. To this end, we analyzed APP CTFs in neurons treated with Aβ-O and/or LY2886721 (LY) [25], a potent BACE1 inhibitor. We found that Aβ-O induced a significant increase in β’-CTF (probably derived from alternative BACE1 cleavage between Tyr10 and Glu11 within the Aβ region) levels, compared to untreated control, which was inhibited by cotreatment with LY (Additional file 3: Figure S3). Aβ-O additionally increased β-CTF levels, although the amounts of β-CTF were much less than those of β’-CTF. These findings support the view that Aβ-O promotes amyloidogenic APP processing. Interestingly, Aβ-O also significantly increased α-CTF levels, which were further augmented by LY treatment (Additional file 3: Figure S3).

## Discussion

In the current study, we investigated whether Aβ augments BACE1 expression in neurons and the associated mechanisms. For this purpose, we established an experimental model in which primary cortical neurons were treated with relatively low concentrations of Aβ-O or Aβ-F for relatively long periods that facilitated analysis of cellular responses without obvious cell death, mimicking the pathological conditions of AD. Using this model, we initially showed that Aβ-O treatment induces a significant increase in BACE1 protein levels while Aβ-F produces only a marginal effect. In contrast, APP and ADAM10 levels remained unaltered, clearly indicating a specific effect of Aβ-O on BACE1. Our findings are consistent with previous studies describing Aβ42-induced BACE1 upregulation [16, 26, 27]. For example, Sadleir and Vassar [16] reported that treatment of mouse primary neurons with 5–10 μM Aβ-O for 1–2 days enhanced BACE1 protein levels accompanied by elevation of APP. The differential responses of APP expression to Aβ-O may be attributed to the species differences of the neurons employed.

Next we focused on the cellular mechanisms by which Aβ-O elevates BACE1 protein expression. BACE1 mRNA levels were unaltered by Aβ-O, indicating that elevation is not regulated at the transcriptional level. Several studies have demonstrated stimulation of BACE1 transcription upon Aβ-O treatment in cultured cells other than primary neurons [19, 26, 28]. In contrast, Sadleir and Vassar [16] showed no effects of Aβ-O on BACE1 mRNA levels in mouse primary neurons. These discrepant findings may be attributed to the different cell types or experimental conditions employed. We further examined whether Aβ-O acts on post-transcriptional regulation. Interestingly, analysis of cellular responses to Aβ-O revealed time-dependent induction of both eIF2α phosphorylation and caspase-3 activation. Recent studies have indicated that activated eIF2α increases BACE1 translation under specific conditions, such as energy deprivation, oxidative stress and viral infection, although eIF2α activation generally elicits translational attenuation [22, 29, 30]. However, data from the current study showed that Aβ-O augments the levels of exogenously expressed BACE1, which is independent of p-eIF2α-mediated translational control, suggesting that Aβ-O regulates BACE1 protein expression not at the translational, but the post-translational level. Consistently, Sadleir et al. [27] reported genetic evidence that p-eIF2a is not responsible for the BACE1 increase in the mouse model of AD. While endogenous BACE1 was elevated on day 2 following Aβ-O treatment, we observed an increase in exogenous BACE1 only on day 3. This difference may be due to the gradual increase in expression of exogenous BACE1 by day 3 masking the enhancement of BACE1 by Aβ-O via a post-translational mechanism.

**Fig. 5:**
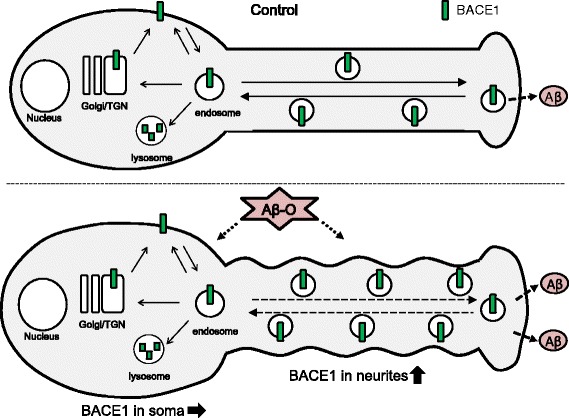
A schema illustrating the mechanism by which Aβ oligomers (Aβ-O) induce BACE1 elevation in neurons. BACE1 levels in neurites, but not soma, are specifically increased in Aβ-O-treated neurons, compared with untreated control. Aβ-O possibly impairs the trafficking of BACE1 in neurites, leading to reduced transport to lysosomal compartments and augmentation in neurites. Aberrantly increased BACE1 consequently promotes Aβ production

Taken together, the data strongly suggest that Aβ-O elevates BACE1 levels via a post-translational mechanism in our neuron model. To gain further insights into the mechanism of Aβ-O-mediated BACE1 upregulation, immunocytochemical analysis was performed to investigate the subcellular localization of BACE1. Intriguingly, Aβ-O treatment induced a significant increase in BACE1 immunoreactivity in neurites, but not soma, while the immunoreactivity of APP remained unaltered. Consistent with our findings, BACE1 is reported to accumulate in dystrophic neurites surrounding amyloid plaques in brains of AD patients and AD model mice [15, 23]. The finding that BACE1 immunoreactivity is enhanced specifically in neurites appears relevant for Aβ-O-mediated BACE1 elevation, as discussed below. The transport mechanism of BACE1 in neurites is poorly understood, although a few proteins with potential roles in the retrograde transport of BACE1 have been identified [31, 32]. We assume that Aβ-O impairs BACE1 transport in axons and dendrites via an as yet unknown mechanism, leading to reduced transport of BACE1 to lysosomal compartments and its augmentation in neurites. In fact, this hypothesis is corroborated by our finding that chloroquine treatment induces a similar increase in BACE1 immunoreactivities in neurites, although there is some difference between the effects of Aβ-O and chloroquine on BACE1. Consistently, a previous study reported that Aβ42 induces upregulation of BACE1 and downregulation of Uch-L1, the latter of which interferes with BACE1 lysosomal degradation in neuronal cells [33]. Stagnation of BACE1 in neuronal processes may increase the probability of convergence between BACE1 and APP [34], as implied by our preliminary data (Additional file 1: Figure S1), possibly leading to enhancement of amyloidogenic APP processing and Aβ generation (Fig. 5). A few molecules are involved in BACE1 degradation, among which GGA3 has been most extensively investigated [14, 24, 32, 33]. GGA3 appears to participate in BACE1 sorting from endosomes to lysosomes, and caspase-mediated depletion of GGA3 stabilizes BACE1 [14, 35]. One hypothesis is that Aβ affects GGA3. However, our preliminary data indicate that Aβ-O does not influence GGA3 levels (data not shown). BACE1 sorting between the plasma membrane, endosomes and TGN appears to be regulated by sorting-associated molecules, such as GGA1, sorting nexin 6 and sortilin [36–38]. Another possibility is that Aβ-O causes dysregulation of BACE1 sorting, resulting in its augmentation. Further studies taking neuronal polarity into consideration are necessary to elucidate the mechanism of Aβ-O-induced BACE1 elevation.

In our experiments, treatment with Aβ-O, but not Aβ-F, led to activation of the eIF2α pathway and the apoptosis cascade. We observed a significant time-dependent increase in cleaved caspase 3 in Aβ-O-treated neurons, consistent with the findings of numerous previous studies [16, 39]. However, despite a marked increase in activated caspase 3, limited cell death was observed in our experimental model. It is possible that Aβ-O-induced caspase activation is not directly associated with cell death, which may have resulted from the lower Aβ-O concentration used in our experiments. In fact, earlier studies have shown that activation of caspase 3 does not necessarily correspond to cell death in AD brain [40, 41]. P-eIF2α was additionally induced upon Aβ-O treatment. Consistently, p-eIF2α is reported to increase in the brains of AD patients and AD model mice [42, 43]. Among the four eIF2α kinases (PERK, PKR, GCN2, HRI), PERK is known to be activated by ER stress [44], which is implicated in AD pathology [45–47]. The PERK pathway may play a major role in the activation of eIF2α in AD model mice [43, 48]. However, the ER stress marker protein GRP78 was not increased in Aβ-O-treated neurons in our study. Furthermore, protein levels of GRP78 and p-eIF2α were increased while those of BACE1 and APP were decreased in neurons treated with the ER stress inducer, thapsigargin. Based on these findings, we propose that typical ER stress does not participate in cellular responses to Aβ-O in our experimental model.

We have presented evidence that oligomeric Aβ upregulates BACE1 via a post-translational mechanism involving its altered subcellular localization. Our data also suggest that Aβ oligomers modulate amyloidogenic APP processing. The present findings imply the direct involvement of Aβ oligomers in BACE1 elevation in brains of AD patients and AD model mice. A vicious cycle is proposed to exist whereby soluble Aβ oligomers augment Aβ production by promoting amyloidogenic processing of APP through BACE1 modulation [15, 19]. Thus, Aβ oligomers contribute to the progression of AD pathology, not only through its neurotoxicity and synaptotoxicity but also the cycle of Aβ production via a feed-forward mechanism. Termination of this vicious cycle may therefore present an effective approach to prevent pathological progression of AD. To this end, therapeutic options targeting Aβ oligomers or BACE1 appear promising. Furthermore, the post-translational mechanisms underlying oligomeric Aβ-induced increase in BACE1 in neurons remain to be established. Our neuron model system provides a useful tool to resolve these critical issues associated with AD pathology.

## Conclusion

We used a neuron model system to investigate the effects of Aβ-O and Aβ-F on BACE1 expression as well as the underlying mechanisms. The present findings collectively demonstrated that Aβ-O induces a significant and specific increase in protein levels of BACE1 via a post-translational mechanism. Immunocytochemical analysis further revealed that Aβ-O affects the subcellular distribution of BACE1 to augment its levels in neuritic compartments, which appears relevant for Aβ-O-mediated BACE1 elevation. We additionally found that Aβ-O induces activation of eIF2α and caspase 3, with no changes in GRP78, indicating that typical ER stress does not participate in cellular responses to Aβ-O in our experimental model. Thus, a vicious cycle appears to exist whereby soluble Aβ oligomers promote Aβ production through post-translational modulation of BACE1, contributing to the pathogenetic mechanism of AD.

## Methods

### Cell culture

Primary cerebral cortical neurons were obtained from 17 day-old embryos of a Wistar rat as described previously [21, 49]. Neurons were plated on poly-L-lysine-coated plates or dishes at a density of 680 cells/mm^2^. Cells were maintained in a humidified atmosphere of 5 % CO_2_/95 % air in Macs Neuro Medium (Miltenyi Biotec, Auburn, CA, USA) containing 0.5 mM L-glutamine, NeuroBrew-21 (Miltenyi Biotec), and penicillin-streptomycin. Half of the medium was replaced with fresh medium every 3–4 days.

### Antibodies

The following primary antibodies were used in this study: anti-Aβ (82E1, IBL, Gunma, Japan; 6E10,Covance, Emeryville, CA, USA), anti-BACE1 (AB5832, Merck Millipore, Darmstadt, Germany; D10E5, Cell Signaling, Danvers, MA, USA), anti-APP (R37 [50]; 22C11, Merck Millipore), anti-ADAM10 (Sigma, St Louis, MO, USA), anti-phospho-eIF2α (Ser51) (Cell Signaling), anti-eIF2α (Assay Biotechnology, Sunnyvale, CA, USA), anti-cleaved caspase 3 (Asp175) (Cell Signaling), anti-GRP78 (BD Biosciences, San Jose, California, USA), anti-β-actin (Sigma), anti-MAP2 (Merck Millipore), and antibody to the rhodopsin tag (1D4) [51] obtained from University of British Columbia.

### Aβ preparation and treatment

Aβ42 oligomers and fibrils were prepared as described previously [20, 52]. Briefly, human Aβ(1–42) peptide (Peptide Institute, Osaka, Japan) was dissolved in 1,1,1,3,3,3-hexafluoro-2-propanol (HFIP; Sigma) in a chemical fume hood to obtain 1 mM solution. HFIP was evaporated overnight in the hood and further under vacuum for 1 h, and dried peptide films stored at −30 °C. Prior to use, dried Aβ peptide was resolved in DMSO to prepare 5 mM stock, and sonicated in an ultrasonic bath sonicator for 10 min. To prepare oligomers, 5 mM Aβ DMSO stock was diluted in DMEM/F12 and left for 1 day at 4 °C. For preparation of fibrils, Aβ stock was diluted in 0.1 M Tris (pH 7.4) and shaken for 2 days at room temperature. Immediately before addition to neurons (9 days *in vitro* (DIV)), Aβ preparations were diluted in regular medium and used to replace the entire medium. Control cultures were treated with the same concentration of DMSO.

### Recombinant adenoviruses

Recombinant adenoviruses expressing BACE1 were prepared using an Adenovirus Dual Expression Vector Kit (Takara Bio, Shiga, Japan) as described previously [21]. In recombinant adenoviruses, human BACE1 cDNA with a C-terminal rhodopsin tag [53, 54] was expressed under the CAG promoter. To evaluate the effect of Aβ-O on exogenous BACE1, primary neurons were infected with recombinant BACE1 adenoviruses at DIV8. One day after adenovirus infection, neurons were treated with Aβ-O as described above, and maintained for 1–3 days.

### Cell survival assay

Primary cortical neurons cultured on 12-well plates were treated with Aβ-O, Aβ-F or vehicle for 2 or 3 days. Cell Counting Kit-8 solution (Dojindo, Kumamoto, Japan) was added to each well, and the plates left in a CO_2_ incubator for 2 h. Absorbance was measured at 450 nm using a microplate reader. Absorbance of the medium was subtracted as a blank from that of each sample.

### Western blot analysis

Cells were lysed on ice in RIPA buffer (10 mM Tris pH 8.0, 150 mM NaCl, 1%NP-40, 0.5 % sodium deoxycholate, 0.1 % SDS, 5 mM EDTA) containing protease inhibitors (aprotinin, leupeptin, pepstatin, PMSF) and phosphatase inhibitors (NaF, Na_3_VO_4_). After rocking for 1 h at 4 °C, samples were centrifuged at 100,000 x *g* for 30 min, and the supernatants used as cell lysates. The protein content in cell lysates was estimated with the bicinchoninic acid assay (Pierce, Rockford, IL, USA). Samples containing equal amounts of protein were mixed with 2x Laemmli sample buffer and incubated at 95 °C for 3 min, followed by separation on 9 or 12 % polyacrylamide gels and transfer to polyvinylidene difluoride (PVDF) membranes. Blots were blocked in 5 % non-fat dried milk in phosphate-buffer saline (PBS) containing 0.05 % Tween-20, and probed with primary antibodies, followed by secondary peroxidase-labeled anti-rabbit or mouse IgG. The Can Get Signal Immunoreaction Enhancer Solution (Toyobo, Osaka, Japan) was occasionally incubated with primary antibodies to enhance immunoreaction. Protein expression was detected with a chemiluminescence reagent (Perkin-Elmer, Boston, MA, USA), and the resulting images examined with a LAS-1000 (Fuji Film, Tokyo, Japan) image analyzer. β-Actin was used as the internal control to normalize the levels of proteins of interest.

### Analysis of APP CTFs

APP CTFs were analyzed by immunoprecipitation-Western blotting, as described previously [21]. Briefly, samples containing an equal amount of protein were immunoprecipitated with anti-APP antibodies (R37) and protein G-agarose at 4 °C overnight. The immunoprecipitated materials were washed, eluted in 2 x Tris/Tricine sample buffer, and subjected to Tris/Tricine SDS-PAGE, followed by Western blot analysis with anti-APP (R37).

### Semi-quantitative reverse transcription (RT)-PCR

Semi-quantitative RT-PCR was essentially performed using a previously documented protocol [55]. Briefly, total RNA was extracted from cells using the Gene Elute Mammalian Total RNA Miniprep Kit (Sigma). RT was performed in a total reaction volume of 20 μl containing 1 μg total RNA and 25 μg/ml oligo (dT)15 using the Improm II Reverse Transcription system (Promega, Madison, WI, USA), according to the manufacturer’s instructions. For semi-quantitative RT-PCR, amplification was performed using 1 μl RT reaction mixture in the presence of 200 μM dNTPs, 0.5 μM primers, and 1 μl Advantage 2 Polymerase mix (Stratagene, La Jolla, CA, USA) in a final volume of 50 μl. The primer pairs used were: 5’-ATTCCCTATACACTGGCAGTC-3’ and 5’-CCATGACATAGGCTATGGT-3’ for BACE1, and 5’-GCAGGAGCTGAATGACCGCT-3’, and 5’-CGGTGAGGTCAGGCTTGGAA-3’ for vimentin. The following PCR conditions were employed: 27 cycles at 95 °C for 1 min, 60 °C for 1 min, and 72 °C for 1 min for BACE1, and 25 cycles at 95 °C for 1 min, 64 °C for 1 min, 72 °C for 1 min for vimentin, used as an internal control. Amplification products were separated on 0.9 % agarose gels, visualized using ethidium bromide staining, and quantified with an LAS-1000 image analyzer. Under the above PCR conditions, band intensities of amplified products were proportional to the amounts of cDNA used in the reaction, confirming the validity of our analysis.

### Immunocytochemistry

Immunocytochemical analysis was essentially performed as described previously [24]. Primary neurons cultured on coverslips were fixed with 4 % paraformaldehyde in PBS. Fixed cells were permeabilized and blocked with 0.3 % Triton X-100 and 1 % horse serum in PBS, and incubated with primary antibody for BACE1 (D10E5) or APP (22C11) for 1 h, followed by Alexa 488-conjugated anti-rabbit IgG (Molecular Probes, Eugene, OR, USA) for 1 h. For double immunofluorescence staining, cells were subsequently stained with anti-MAP2 antibody and Alexa568-conjugated anti-mouse IgG (Molecular Probes). Specimens were examined under an LSM 780 laser scanning confocal microscope (Carl Zeiss, Jena, Germany). The fluorescence intensities of neurites and soma were quantified according to previously documented methods [56]. Briefly, to quantify fluorescence intensity, 1 pixel-wide line segments were traced along 50 μm of neurites with soma as the starting point. The mean fluorescence intensity in soma was quantified by drawing a region around the soma. For each condition, ~20 cells from two different cultures were analyzed. To distinguish axons and dendrites, cells doubly immunostained with anti-BACE1 and anti-MAP2 were analyzed as above; an example image is shown in Additional file 4: Figure S4.

### Statistical analysis

All results are presented as means ± SEM. Data were statistically analyzed using one-way analysis of variance (ANOVA), followed by Bonferroni’s multiple comparison test or Student’s *t*-test with a significance threshold of *p* < 0.05.

## Additional files

Additional file 1: Figure S1. Double immunofluorescence staining with BACE1 and APP antibodies. Primary cortical neurons were treated with 2.5 μM Aβ-O or vehicle for 3 days, followed by double immunofluorescence staining with anti-BACE1 and anti-APP antibodies. Scale bar = 20 μm. (PDF 384 kb) Additional file 2: Figure S2. Effects of chloroquine on BACE1 levels and immunoreactivities in primary neurons. (**A**) Primary neurons were treated with or without 25 μM chloroquine (CQ) for 1 day, followed by Western blot analysis with anti-BACE1. Relative levels of BACE1 were quantified and graphed. Data represent means ± SEM of three samples from two separate experiments. **p* < 0.05, compared with control. (**B**) Primary cortical neurons treated with or without 25 μM CQ for 1 day were immunostained with anti-BACE1. Scale bar = 20 μm. (**C**) Fluorescence intensities of BACE1 in soma and neurites in (**B**) were separately quantified as described in Methods, and the relative levels depicted on a graph. (*n* = 24, ****p* < 0.001). (PDF 1277 kb) Additional file 3: Figure S3. Analysis of APP CTFs in primary neurons. Primary neurons were treated with either vehicle, 1 μM LY2886721 (LY) (Selleck Chemicals, Houston, TX, USA), 2.5 μM Aβ-O, or Aβ-O plus LY for 3 days. APP CTFs were analyzed as described in Methods. Relative levels of β’-CTF and α-CTF were quantified and graphed. Data represent means ± SEM from three separate experiments. ***p* < 0.01, compared with control. (PDF 59 kb) Additional file 4: Figure S4. Quantification of fluorescence intensities in axons and dendrites. After double immunofluorescent staining of primary neurons with anti-BACE1 (green) and anti-MAP2 (red) antibodies, specimens were examined under a LSM780 microscope. BACE1 fluorescence intensities along MAP2-positive dendrites (red line) and MAP2-negative axons (blue line) were quantified as described in Methods. Scale bar = 10 μm. (PDF 66 kb)

## Abbreviations

AD: Alzheimer’s disease
Aβ: Amyloid β-protein
Aβ-O: Aβ42 oligomers
Aβ-F: Aβ42 fibrils
APP: Amyloid precursor protein
CTF: C-terminal fragment
ER: Endoplasmic reticulum
PBS: Phosphate-buffered saline
p-eIF2α: Phosphorylated eIF2α
