# Supplementary figures and images for: Amyloid β-protein oligomers upregulate the β-secretase, BACE1, through a post-translational mechanism involving its altered subcellular distribution in neurons
Source: Mol Brain. 2015 Nov 9;8:73. doi: 10.1186/s13041-015-0163-5 (PMC4638102; doi:10.1186/s13041-015-0163-5)

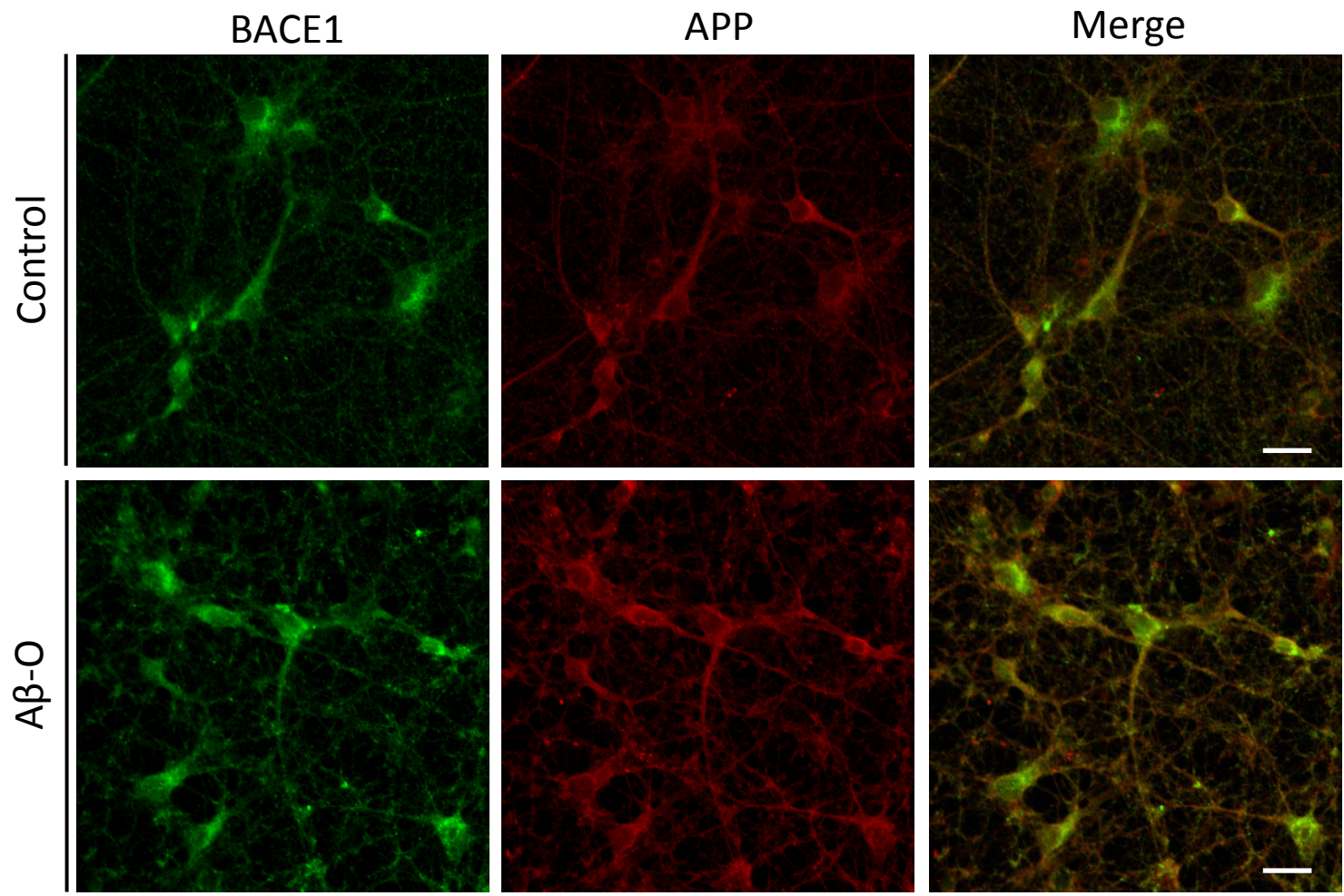

Supplement: Additional file 1: Figure S1. — Double immunofluorescence staining with BACE1 and APP antibodies. Primary cortical neurons were treated with 2.5 μM Aβ-O or vehicle for 3 days, followed by double immunofluorescence staining with anti-BACE1 and anti-APP antibodies. Scale bar = 20 μm. (PDF 384 kb) [file 13041_2015_163_MOESM1_ESM.pdf]

A

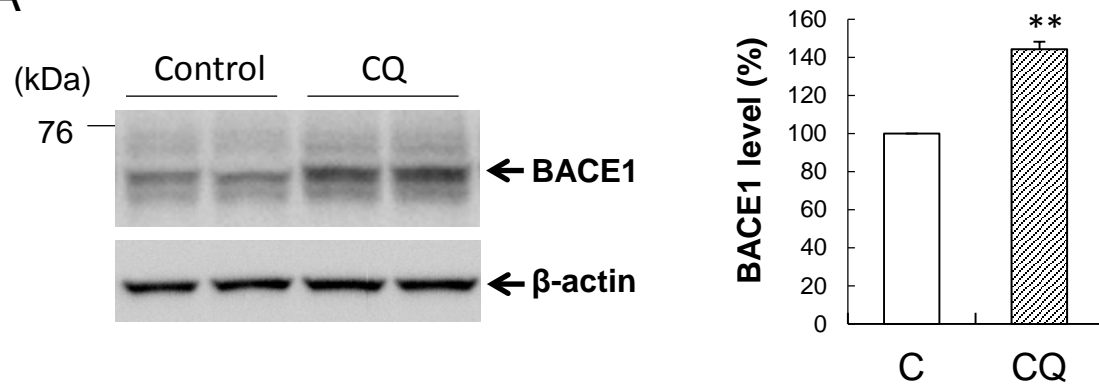

B

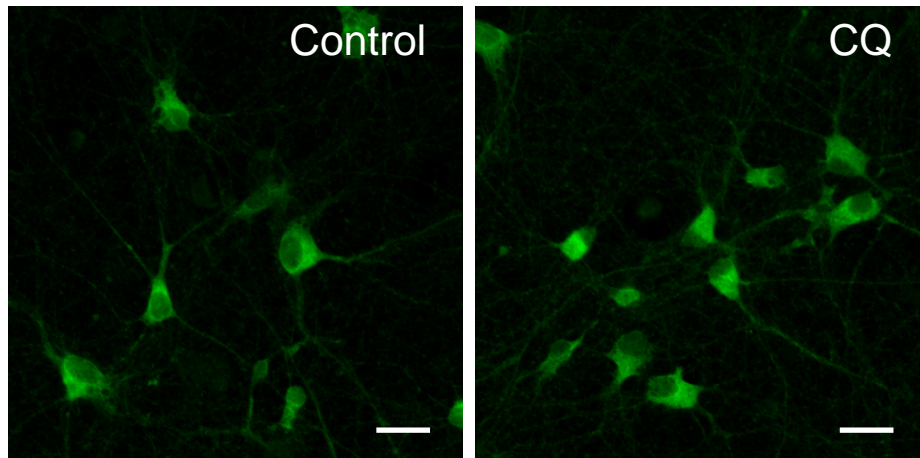

C

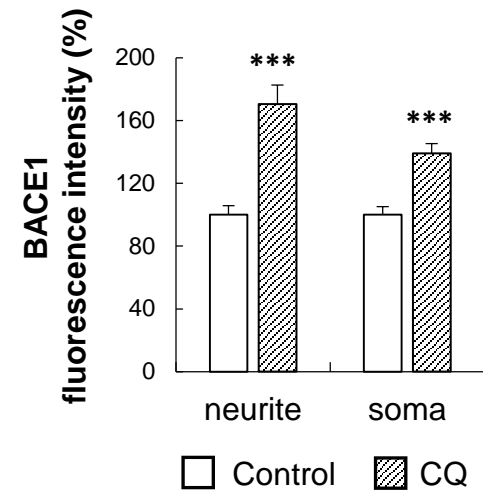

Supplement: Additional file 2: Figure S2. — Effects of chloroquine on BACE1 levels and immunoreactivities in primary neurons. (A) Primary neurons were treated with or without 25 μM chloroquine (CQ) for 1 day, followed by Western blot analysis with anti-BACE1. Relative levels of BACE1 were quantified and graphed. Data represent means ± SEM of three samples from two separate experiments. *p < 0.05, compared with control. (B) Primary cortical neurons treated with or without 25 μM CQ for 1 day were immunostained with anti-BACE1. Scale bar = 20 μm. (C) Fluorescence intensities of BACE1 in soma and neurites in (B) were separately quantified as described in Methods, and the relative levels depicted on a graph. (n = 24, ***p < 0.001). (PDF 1277 kb) [file 13041_2015_163_MOESM2_ESM.pdf]

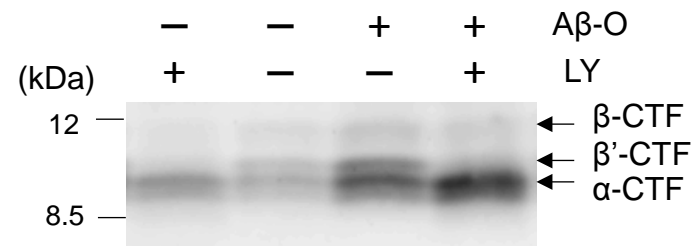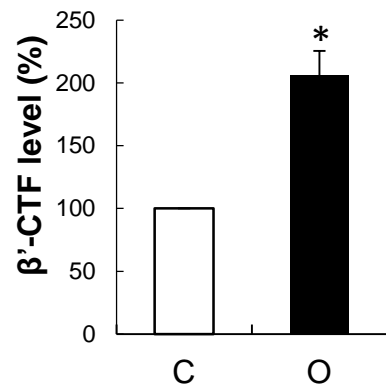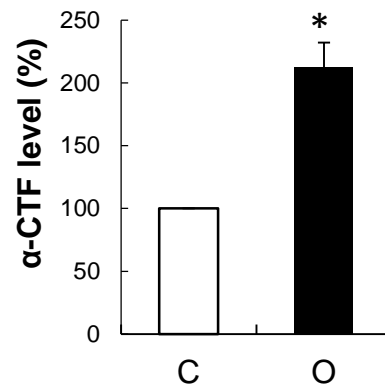

Supplement: Additional file 3: Figure S3. — Analysis of APP CTFs in primary neurons. Primary neurons were treated with either vehicle, 1 μM LY2886721 (LY) (Selleck Chemicals, Houston, TX, USA), 2.5 μM Aβ-O, or Aβ-O plus LY for 3 days. APP CTFs were analyzed as described in Methods. Relative levels of β’-CTF and α-CTF were quantified and graphed. Data represent means ± SEM from three separate experiments. **p < 0.01, compared with control. (PDF 59 kb) [file 13041_2015_163_MOESM3_ESM.pdf]

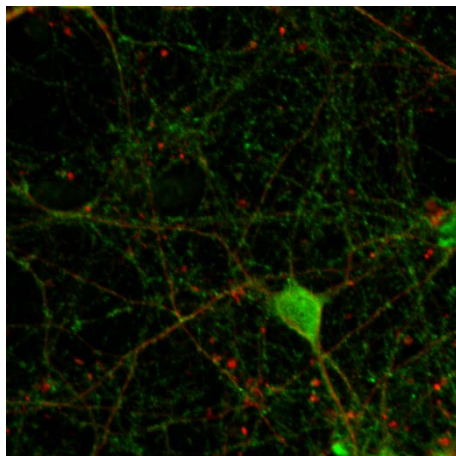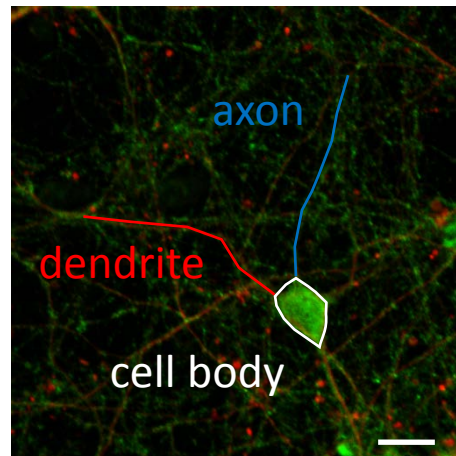

Supplement: Additional file 4: Figure S4. — Quantification of fluorescence intensities in axons and dendrites. After double immunofluorescent staining of primary neurons with anti-BACE1 (green) and anti-MAP2 (red) antibodies, specimens were examined under a LSM780 microscope. BACE1 fluorescence intensities along MAP2-positive dendrites (red line) and MAP2-negative axons (blue line) were quantified as described in Methods. Scale bar = 10 μm. (PDF 66 kb) [file 13041_2015_163_MOESM4_ESM.pdf]
